# Pilocytic astrocytoma and glioneuronal tumor with histone H3 K27M mutation

**DOI:** 10.1186/s40478-016-0361-0

**Published:** 2016-08-12

**Authors:** Cordelia Orillac, Cheddhi Thomas, Yosef Dastagirzada, Eveline Teresa Hidalgo, John G. Golfinos, David Zagzag, Jeffrey H. Wisoff, Matthias A. Karajannis, Matija Snuderl

**Affiliations:** 1 NYU School of Medicine, New York, NY 10016 USA; 2 Department of Pathology, NYU Langone Medical Center, 550 1st Ave, New York, NY 10016 USA; 3 Department of Neurosurgery, NYU Langone Medical Center, 550 1st Ave, New York, NY 10016 USA; 4 Division of Pediatric Hematology/Oncology, Departments of Pediatrics and Otolaryngology, NYU Langone Medical Center, 550 1st Ave, New York, NY 10016 USA

**Keywords:** Glioblastoma, Histone H3 K27M, Pilocytic astrocytoma, Glioneuronal tumor

Pediatric glioblastoma (GBM) can be sub-classified into several molecular subgroups, including a group defined by a mutation in histone H3 at position amino acid 27 resulting in the replacement of lysine by methionine (K27M) [1]. K27M GBMs are located in the midline, often occur in children, and have been shown to have worse prognosis than other GBM subgroups, with a median survival of 6 months [2–5]. The 2016 revision of the World Health Organization (WHO) Classification of Tumors of the Central Nervous System now recognizes “diffuse midline glioma, H3 K27M-mutant” as a distinct clinic-pathological entity and recommends grading as Grade 4 [5]. A robust immunohistochemical stain has been recently developed to specifically detect the K27M mutation. It can be used to help diagnose these high-grade tumors, particularly in the setting of a small biopsy where the amount of tissue is insufficient for molecular studies [6]. Here we report two patients with midline pilocytic astrocytoma and a glioneuronal tumor, respectively, harboring a K27M mutation. Our findings show that K27M mutations may be present in a spectrum of brain tumors with less aggressive clinical behavior and prolonged survival. Our data indicates that the entity of H3 K27M-mutant tumors may represent a spectrum including not only diffuse gliomas [5] but also less aggressive tumors than previously recognized, and that histone H3 K27M mutation should not be used as the sole criterion for the diagnosis of WHO Grade IV and to imply a dismal prognosis and aggressive management.

Patient 1 had a past medical history significant for migraines and presented in July, 2012 after head trauma. A computed tomography (CT) scan at the time showed a lesion (Fig. 1a), and was followed by imaging. Three years later, at 27 years of age, she presented with diffuse headaches, nausea, and blurry vision. On examination she was noted to have papilledema, and imaging demonstrated an expansile enhancing mass lesion in the posterior, right thalamus (Fig. 1b). At the time of surgery, a well-demarcated border was noted by the neurosurgeon and good plane was obtained that allowed almost gross total resection. Macroscopically the tumor was firm and chalky white suggestive densely gliotic lesion. Microscopically, the tumor showed biphasic pattern with loose piloid areas (Fig. 1e) and dense areas containing numerous eosinophilic granular bodies (Fig. 1f, arrow) and foci of microvascular proliferation. Mitotic figures were rare but the Ki-67 proliferation index was elevated up to 20 % focally (Fig. 1f insert). The tumor was diagnosed as pilocytic astrocytoma WHO Grade I with elevated Ki-67 proliferation index and the possibility of more aggressive biological behavior was raised in the report. Immunohistochemical studies showed that the tumor was positive for K27M mutant protein (Fig. 1h). Copy number analysis using Illumina 450 k Infinium array showed no detectable gains or losses (Fig. 1i). The tumor was negative for 3′ BRAF genomic duplication tested by fluorescence in situ hybridization (FISH) and BRAF V600E by next generation sequencing. No adjuvant therapy was initiated and the lesion was followed until 14 months after surgery, when it showed local recurrence (Fig. 1d). Further resection and adjuvant therapy were recommended.

**Fig. 1:**
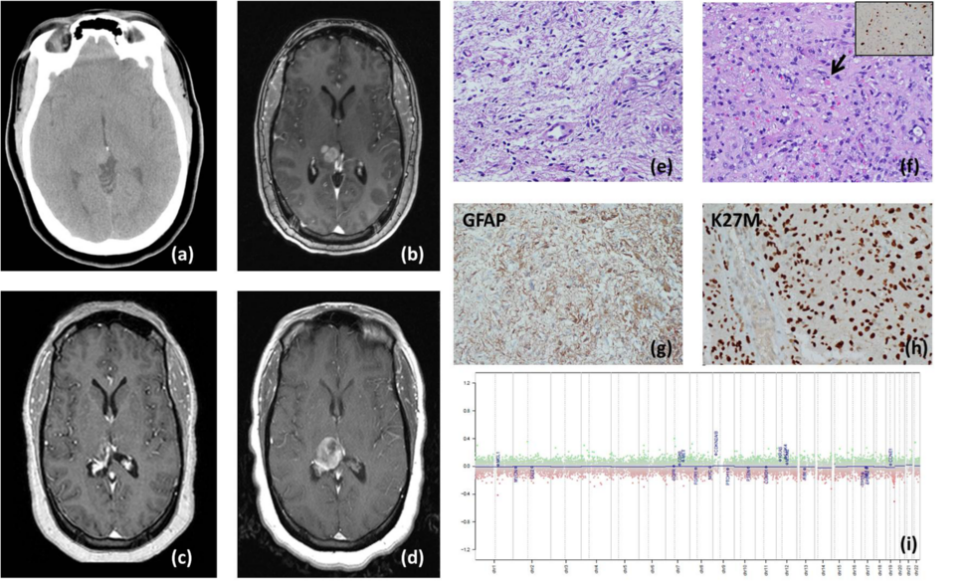
The pathologic and radiologic imaging of Patient 1; **a** axial non-contrast CT 3-years preoperative, showing asymmetry of the posterior thalami; **b** axial T1-weighted contrast-enhanced MRI immediately preoperative showing enhancing mass lesion in the right posterior thalamus; **c** axial T1-weighted contrast-enhanced MRI 8 months postoperative showing resection of the lesion with no residual tumor **d** axial T1-weighted contrast-enhanced MRI 13 months postoperative showing recurrence of the lesion in the right posterior thalamus; **e** histological evaluation shows pilocytic astrocytoma with piloid loose areas and **f** dense areas with eosinophilic granular bodies (**f**, *arrow*). The tumor showed focally elevated Ki-67 up to 10 % (**f**, *insert*); The tumor was strongly positive for GFAP (**g**) and histone H3 K27M (**h**); **i** copy number analysis using Illumina 450 k Infinium array showed no large chromosomal gains or losses of focal amplifications in glioma associated genes (*blue labels*)

Patient 2 was a 10 year-old female with complaints of headaches and vomiting in June, 2014. Imaging showed a heterogeneously enhancing midbrain mass (Fig. 2a, c). There was marked hydrocephalus due to obstruction. She underwent resection and the pathology showed a tumor composed of small round blue cells with high mitotic activity, expressing synaptophysin (Fig. 2). The tumor was overall negative for GFAP; however scattered larger GFAP positive cells were noted (Fig. 2g, insert) and it was deemed not to be possible to distinguish whether they represent reactive astrocytes or tumor cells. Next-generation sequencing of tumor DNA identified mutations in FGFR1 (N546K), NF1 (E1436fs*4, W2317) and histone H3F3A (K27M). The K27M mutation was confirmed in the tissue by immunohistochemistry (Fig. 2h). Copy number analysis using Illumina 450 k Infinium array showed no detectable gains or losses (Fig. 2i). Since the tumor did not fit any WHO entity and due to ambiguous histological features and molecular analysis, the diagnosis was reported descriptively as an anaplastic glioneuronal tumor, WHO grade III. Post-operatively, patient received proton beam radiation therapy with adjuvant chemotherapy with temozolomide (90 mg/m^2^) for 6 weeks. Twenty-two months after surgery she remains stable with no recurrence (Fig. 2b, d).

**Fig. 2:**
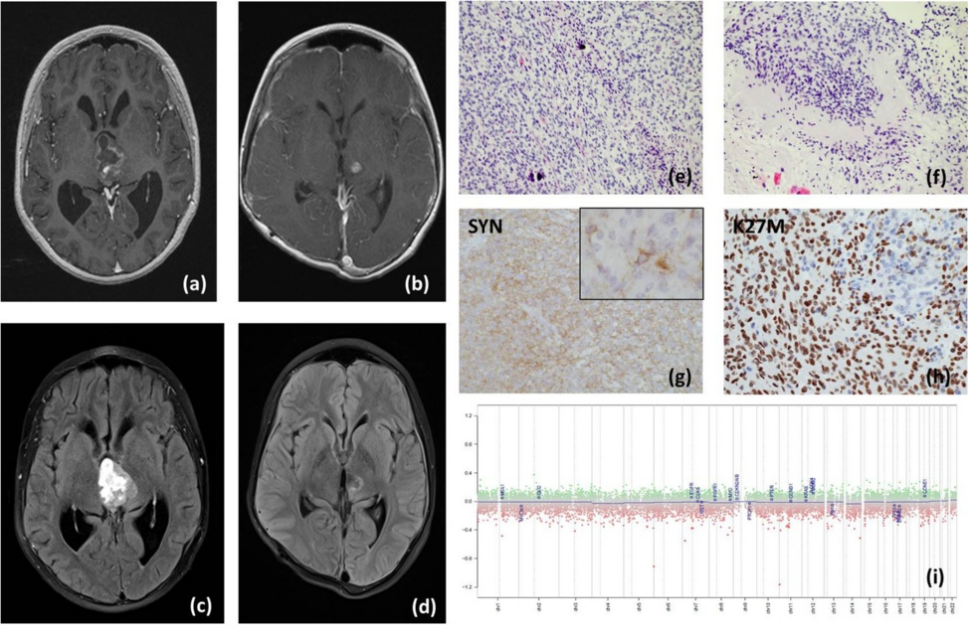
The pathologic and radiologic imaging of Patient 2; **a** axial T1-weighted contrast-enhanced MRI immediately preoperative; **b** axial T1-weighted contrast-enhanced MRI 22 months postoperative showing no residual tumor; **c** axial T2 FLAIR MRI immediately preoperative; **d** axial T2 FLAIR MRI 22 months postoperative showing no residual/recurrent tumor; histological evaluation shows a predominantly small round blue cell tumor tumor (**e**) with neuropil islands and rosettes (**f**); the tumor was strongly positive for neuronal differentiation marker synaptophysin (**g**). While small round blue cells were negative for GFAP, rare scattered larger GFAP positive cells were noted (**g**, *insert*). Tumor was strongly positive for histone H3 K27M (**h**); **i** copy number analysis using Illumina 450 k Infinium array showed no large chromosomal gains or losses of focal amplifications in glioma associated genes (*blue labels*)

While molecular analysis of tumors adds important information, it cannot replace histological assessment. The two patients here were diagnosed with Grade I and Grade III tumors respectively and were both treated with surgical resection and the latter also with radiation therapy and adjuvant chemotherapy. Due to the difficulty in achieving significant resection and high risk of morbidity, patients with midline tumors are often only biopsied for diagnosis. The K27M immunohistochemical marker is often used as a diagnostic tool to confirm a midline high-grade astrocytoma and patients are typically treated with radiation and chemotherapy. However despite aggressive treatment, the 3-year overall survival (OS) of patients with K27M GBMs is 5 %. Therefore many consider identifying the K27M mutation as a strong indication of a high-grade glioma. The cases presented show that the K27M mutation may be present in less aggressive “lower grade” gliomas as well as glioneuronal tumors. Our tumors did not show histological features of diffuse infiltrating glioma/GBM on imaging or histopathology. One possible molecular clue arguing against the diagnosis of GBM in our cases may be a copy number profile without the typical genomic gains or losses seen in GBM (Figs. 1i and 2i). Our report highlights a morphological as well as clinical spectrum of histone H3 K27M mutant tumors. Our findings imply that, especially in cases where only a small biopsy is obtained, a sample positive for H3K27M by immunohistochemistry may be inappropriately diagnosed as a high-grade astrocytoma or even GBM and routinely receive aggressive adjuvant treatment. Therefore, the presence of a histone H3 K27M mutation should be evaluated in the context of the histology and other genomic features to ensure accurate diagnosis. The significance of histone H3 K27M mutations in tumors that are histologically not diffuse gliomas is currently unknown; therefore additional studies are necessary to elucidate the prognostic impact of this molecular alteration.

## Abbreviations

CT, computed tomography; FISH, fluorescence in situ hybridization; GBM, glioblastoma; K27M, mutation in histone 3 of K27 residue; OS, overall survival; WHO, World Health Organization
